# *Porphyromonas gingivalis* and *Treponema denticola* Synergistic Polymicrobial Biofilm Development

**DOI:** 10.1371/journal.pone.0071727

**Published:** 2013-08-26

**Authors:** Ying Zhu, Stuart G. Dashper, Yu-Yen Chen, Simon Crawford, Nada Slakeski, Eric C. Reynolds

**Affiliations:** 1 Oral Health Cooperative Research Centre, Melbourne Dental School, Bio21 Institute, The University of Melbourne, Victoria, Australia; 2 School of Botany, The University of Melbourne, Victoria, Australia; University of North Dakota School of Medicine and Health Sciences, United States of America

## Abstract

Chronic periodontitis has a polymicrobial biofilm aetiology and interactions between key bacterial species are strongly implicated as contributing to disease progression. *Porphyromonas gingivalis, Treponema denticola* and *Tannerella forsythia* have all been implicated as playing roles in disease progression. *P. gingivalis* cell-surface-located protease/adhesins, the gingipains, have been suggested to be involved in its interactions with several other bacterial species. The aims of this study were to determine polymicrobial biofilm formation by *P. gingivalis*, *T. denticola* and *T. forsythia*, as well as the role of *P. gingivalis* gingipains in biofilm formation by using a gingipain null triple mutant. To determine homotypic and polymicrobial biofilm formation a flow cell system was employed and the biofilms imaged and quantified by fluorescent *in situ* hybridization using DNA species-specific probes and confocal scanning laser microscopy imaging. Of the three species, only *P. gingivalis* and *T. denticola* formed mature, homotypic biofilms, and a strong synergy was observed between *P. gingivalis* and *T. denticola* in polymicrobial biofilm formation. This synergy was demonstrated by significant increases in biovolume, average biofilm thickness and maximum biofilm thickness of both species. In addition there was a morphological change of *T. denticola* in polymicrobial biofilms when compared with homotypic biofilms, suggesting reduced motility in homotypic biofilms. *P. gingivalis* gingipains were shown to play an essential role in synergistic polymicrobial biofilm formation with *T. denticola*.

## Introduction

Polymicrobial biofilms are dynamic structures that can alter form or composition based not only on environmental conditions including nutrient supply, shear forces and temperature but also on the synergies and antagonisms between the species that comprise the biofilm and the emergent properties that result from these interactions [1]–[3]. Microbial biofilms are the predominant bacterial lifestyle in habitats where shear forces and bulk phase movement result in removal of unattached bacteria. The oral cavity is a classic example where extensive bacterial biofilms (plaque) can develop on the non-shedding surfaces of teeth and result in the development of polymicrobial plaque-related diseases such as periodontitis [4].

Anaerobic, proteolytic bacterial species including *Porphyromonas gingivalis*, *Treponema denticola* and/or *Tannerella forsythia* are consistently found in elevated numbers in subgingival plaque samples taken from periodontally diseased subjects [5]–[7]. Of the three species, *P. gingivalis* and *T. denticola* are frequently found together in diseased sites [8], [9], while *T. forsythia* may not co-localize spatially with the other two species [10], [11]. Among the three species that are frequently found associated with the clinical measures of chronic periodontitis, *P. gingivalis* has been shown to be a major pathogen, with well-defined virulence factors including cell surface located proteolytic enzymes and adhesins (gingipains) [12], [13].Recent research suggests that a synergistic microbial community is more relevant to disease progression than individual species and it has been suggested that the abilities of micro-organisms to interact with one another are crucial for disease progression [14], [15]. *P. gingivalis* has been shown to exert a community-wide pathogenic influence on the microbiota in an animal model and it has been suggested that the communication of *P. gingivalis* with other inhabitants of subgingival biofilm is crucial for the elevation of pathogenicity and disruption of host immune surveillance [16].

*P. gingivalis* and *T. denticola* displayed synergy in biofilm formation using a static biofilm model, but this was not observed with *T. forsythia* [17], [18]. In dual-species biofilm experiments, *T. denticola* did not form homotypic biofilms, while *P. gingivalis* acted as an initial colonizer of the substratum, enabling subsequent incorporation of *T. denticola* [17], [19]. *P. gingivalis* gingipains and *T. denticola* dentilisin have been shown to be involved in the coaggregation between the two species [19], [20]. All of these studies on *P. gingivalis* and *T. denticola* synergistic biofilm formation used *P. gingivalis* strain 381 or ATCC 33277 in a static biofilm model as *P. gingivalis* W50 is considered to be poor biofilm former [21], [22]. The simplicity of static biofilm system made it a widely used tool in biofilm research, especially for examining early events in biofilm formation [23]. However, its ability to generate mature biofilms is limited due to possible limitations of nutrient supply and lack of bulk phase movement. These limitations in the production of mature biofilms can be addressed using flow chambers and continuous culture systems such as a chemostat [24]. A good example of the application of the later systems is the use of a chemostat to generate *T. denticola* homotypic mature biofilms [25]. We have previously shown that, using a flow cell model, *P. gingivalis* W50 was able to participate in polymicrobial biofilm formation with *T. denticol*a and *T. forsythia* [26]. Although *P. gingivalis* W50 adhered poorly to the glass substratum, with only a few cells attached at the commencement of a constant flow, it managed to proliferate and became the dominant species in the mature polymicrobial biofilm. However, it remains to be elucidated how *P. gingivalis* W50 interacts with the other two species, especially *T. denticola*, and the mechanisms involved in polymicrobial biofilm formation.

In the current study we used a flow cell biofilm model to investigate the ability of *P. gingivalis* and *T. denticola* to form homotypic and polymicrobial biofilms and the role of *P. gingivalis* gingipains in biofilm formation. We demonstrate that both *P. gingivalis* W50 and *T. denticola* ATCC 35405 form extensive mature homotypic biofilms and that there is a strong synergy between the two species in biofilm formation and development. In addition *P. gingivalis* gingipains are essential for biofilm formation and the interactions of *P. gingivalis* with *T. denticola*.

## Methods

### Bacterial Strains and Growth

Bacterial strains used for this study were *Porphyromonas gingivalis* W50, *Treponema denticola* ATCC 35405 and *Tannerella forsythia* ATCC 43037. A gingipain-null mutant of *P. gingivalis* W50 lacking RgpA, RgpB and Kgp (*P. gingivalis* W50ABK) was obtained from the culture collection of the Oral Health Cooperative Research Centre, The University of Melbourne, Australia and was created as described previously [27]. All of the cultures were grown anaerobically at 37°C in a MACS MG500 anaerobic workstation (Don Whitley Scientific, U. K.) containing a gaseous mix of 5% hydrogen, 5% carbon dioxide and 90% nitrogen. *P. gingivalis* was grown in brain heart infusion (BHI), *T. denticola* was grown in oral bacteria growth medium (OBGM), and *T. forsythia* was grown in tryptic soy broth supplemented with 0.3% yeast extract (TSBYK), vitamin K (0.4 μg/mL) and N-acetylmuramic acid (NAM) (10 μg/mL) (Sigma Aldrich, MO, USA) as described previously [26], [28]. Each species was transferred into fresh OBGM to obtain exponential growth. The optical density of the bacterial cultures was adjusted with fresh OBGM to give an absorbance of 2.0 at a wavelength of 650 nm prior to snap freezing in liquid nitrogen and storage at −70°C. OBGM contained brain heart infusion (12.5 g/L), tryptone soya broth (10 g/L), yeast extract (7.5 g/L), sodium thioglycolate (0.5 g/L), asparagine (0.25 g/L) D-glucose (2 g/L), ascorbic acid (2 g/L), sodium pyruvate (1 g/L) and sodium bicarbonate (2 g/L), L-cysteine (1 g/L), ammonium sulfate (2 g/L), thiamine pyrophosphate (6 mg/L), heat inactivated rabbit serum (5% vol/vol), haemin (5 mg/L), menadione (1 mg/L), N-acetylmuramic acid (10 mg/mL) and a volatile fatty acid mix (0.5% vol/vol). All species, including the *P. gingivalis* W50ABK mutant grew well as planktonic cultures in OBGM.

### Flow Cell Preparation

A single track (40 mm long, 16 mm wide and 2 mm deep) was milled into a high-density polyethylene block, serving as the incubation chamber for the flow cell. A standard-sized, uncoated glass microscope coverslip (ProSciTech, QLD, Australia), which served as the attachment substratum for the biofilm, was secured to the flow cell with a silicone adhesive (GE Silicones, General Electric Company, Waterford, NY). Sodium hypochlorite with 0.5% available chlorine was pumped through the flow cell system for 2 h to ensure sterility. This was followed by overnight rinsing with sterile ultrapure water to flush out the bleach. The flow cell system was then treated with pre-reduced 20% OBGM for 2 h at 37°C in an MG500 anaerobic workstation to condition the glass surface with medium prior to inoculation.

### Growth of Biofilm in Flow Cells

Snap frozen stocks of each strain were thawed and used as the inoculum. Inocula for polymicrobial biofilms were first coaggregated by mixing 0.5 mL portions of *P. gingivalis*, *T. denticola* and *T. forsythia* prior to inoculation. After inoculation, the system was incubated for 1 h prior to a constant flow (3 mL/h) of OBGM diluted 4∶1 with water to 20% full strength.

Biofilms adhering to the glass coverslips were harvested 90 h after the commencement of medium flow. They were first rinsed *in situ* with phosphate buffered saline (PBS) to remove culture medium and unattached bacterial cells prior to fixation with 4% paraformaldehyde for 1 h at room temperature. After fixation, residual paraformaldehyde was flushed out with PBS. For subsequent *in situ* analyses by scanning electron microscopy (SEM), the coverslip was removed from the flow cell using a diamond pen. For fluorescence staining, the biofilm was embedded in 20% acrylamide with 0.02% ammonium persulfate and 0.8% *N,N,N*',*N*'-tetramethylethylenediamine (TEMED). The coverslips were then removed and the biofilm embedded in the polymerised acrylamide slab was stored in PBS at 4°C prior to analysis.

### Fluorescent Staining of Biofilms

Biofilms were fluorescently stained essentially as described [26]. Single-species biofilms were stained with Syto 9 DNA dye (6 µM, Life Technologies, Grand Island, NY), and polymicrobial biofilms were subjected to fluorescent *in situ* hybridisation (FISH) using species-specific probes.

### Confocal Laser Scanning Microscopy (CLSM) and Image Analysis

Fluorescently labelled biofilms were visualised on a confocal laser scanning microscope as described previously [26]. The confocal datasets, 5 image stacks in random positions from each of two biological replicates, were analysed with COMSTAT software to determine biometric parameters of the biofilm [29]. The biometric data were statistically analysed using independent t-test and a *P* value of <0.05 was considered to be statistically significant. Three-dimensional reconstructed images were produced using Zeiss LSM image browser (Carl Zeiss, Germany).

### Scanning Electron Microscopy (SEM)

Biofilm samples for SEM were prepared as described [26] and imaged with a Philips XL30 field-emission scanning electron microscope (Philips, Eindhoven, Netherlands) at a voltage of 2 kV.

## Results

### Synergistic Biofilm Formation by *P. gingivalis* and *T. denticola*

**Figure 1:**
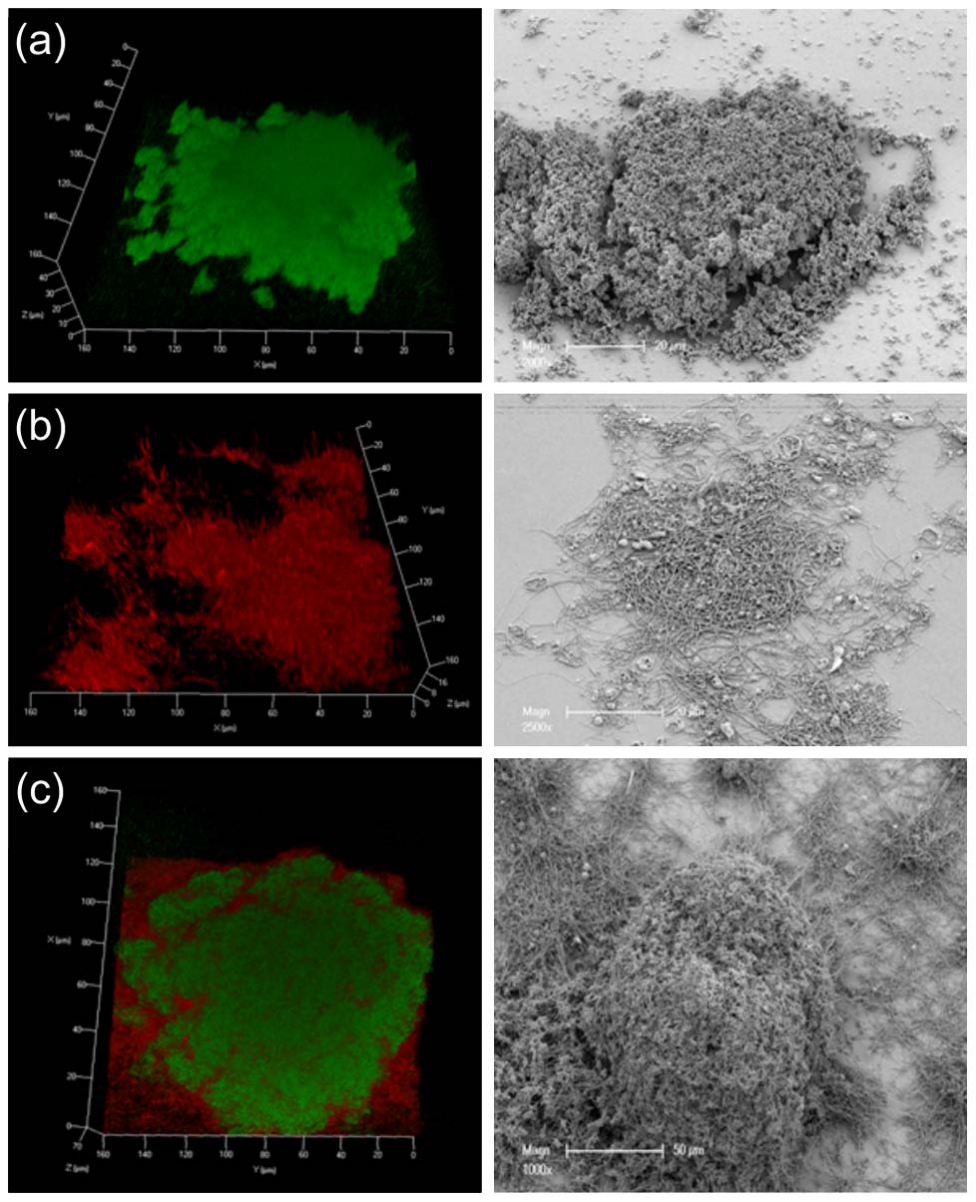
Representative images. Representative images of mature homotypic and polymicrobial biofilms involving *P. gingivalis*, *T. denticola* and *T. forsythia*. Biofilm images were taken 90 h after inoculation of a flow cell. (a) *P. gingivalis* homotypic biofilm (b) *T. denticola* homotypic biofilm (c) Polymicrobial biofilms. Bacterial cells were stained with species-specific FISH probes (red, *T. denticola*; green, *P. gingivalis*). 3D CSLM images are on the left and SEM images are on the right.

Homotypic biofilms of *P. gingivalis* W50 and *T. denticola* were harvested from the flow cell 90 h after the commencement of constant medium flow. Both *P. gingivalis* W50 and *T. denticola* ATCC 35405 formed mature biofilms, characterised by a heterogeneous architecture consisting of microcolonies surrounded by open areas with more scattered colonisation, as revealed by CLSM and SEM (Figure 1a and 1b). The structures of *P. gingivalis* and *T. denticola* homotypic biofilms were quantified from CLSM images taken at five randomly chosen positions each containing a single microcolony from two biological replicates (Table 1). The biovolume and substratum coverage of the *P. gingivalis* W50 biofilms were more than three times greater than *T. denticola* biofilms (Table 1), suggesting *P. gingivalis* has a greater potential for forming homotypic biofilms. The maximum thickness of *P. gingivalis* biofilms was comparable to that of *T. denticola*, however, the average thickness of *P. gingivalis* W50 biofilms was more than double, indicating that *P. gingivalis* biofilms have a greater cell density (Table 1).

**Table 1:** Biometric parameters of *P. gingivalis* and *T. denticola* homotypic biofilm and polymicrobial biofilm formed by *P. gingivalis* W50 ABK, *T. denticola* and *T. forsythia* harvested at 90 h.

	Homotypic biofilms		Polymicrobial biofilm with *P. gingivalis* W50ABK
Biometric parameters	*P. gingivalis* W50	*T. denticola*	*T. denticola*
Biovolume (µm^3^/µm^2^)	3.63±0.36*	0.94±0.62	1.40±0.33
Average thickness of biofilm (µm)	4.93±0.51*	2.25±1.27	4.48±2.94
Maximum thickness of biofilm (µm)	29.66±8.09	25.67±6.13	36.64±14.04
Substratum coverage (%)	28.42±1.71*	6.06±6.57	6.48±0.94

Data are expressed as means ± standard deviations of two biological replicates, from five CLSM images at random positions from each biological replicate. All images were analysed using COMSTAT software.

* Significantly different *to T. denticola* homotypic biofilm values, as determined by Students' T-test (p≤0.05).

**Figure 2:**
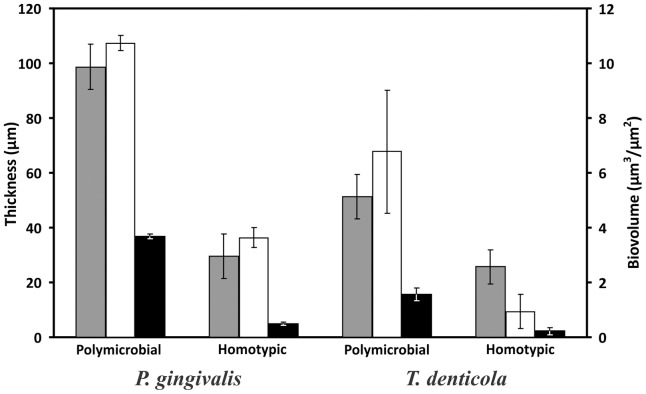
Synergistic *P. gingivalis* and *T. denticola* biofilm formation. Both polymicrobial and homotypic biofilms were produced using a flow cell under identical conditions. The two sets of bars above the species name refer to the biometric parameters measured using the species-specific fluorescent probe for the species grown either as part of the polymicrobial biofilm or as a homotypic biofilm. The primary vertical axis (left) is for maximum (grey bars) and average (black bars) biofilm thickness (μm) and the secondary vertical axis (right) is for biovolume (white bars) (μm^3^/μm^2^). Data are expressed as means ± standard deviations of five CLSM images at random positions from biological replicates. All images were analysed using COMSTAT software.

Polymicrobial biofilms formed by *P. gingivalis* W50, *T. denticola* and *T. forsythia* consisted of large microcolonies (Figure 1c). *T. forsythia* was present in extremely low numbers and was only detected on close examination of the biofilms (data not shown). *T. denticola* formed the basal layer of the mature polymicrobial biofilms, while *P. gingivalis* was the dominant species of the microcolonies (Figure 1c). *T. denticola* was closely associated with *P. gingivalis* in microcolonies, but its abundance decreased towards the top of the microcolonies (further away from the substratum). *P. gingivalis*, *T. denticola* and *T. forsythia* polymicrobial biofilms showed significantly higher colonisation of the substratum compared with either *P. gingivalis* or *T. denticola* alone (Figure 2). There was an approximately three-fold increase in total biovolume for *P. gingivalis* and a six-fold increase for *T. denticola* in polymicrobial microcolonies compared with single-species biofilms (Figure 2). The thickness of biofilms for each species in microcolonies also increased dramatically (Figure 2). The total biovolume and average thickness of the two species together in polymicrobial biofilms increased by approximately four and seven-fold, respectively, when compared with the sum of each species in homotypic biofilms, suggesting a strong synergy in biofilm formation between these species. Moreover, the morphology of *T. denticola* in single-species biofilms (Figure 1b) was distinctly different from that in the polymicrobial biofilm (Figure 1c). In single-species biofilms, the vast majority of *T. denticola* cells did not display the spiral morphology that is characteristic of spirochetes. In contrast, in the polymicrobial biofilms, *T. denticola* cells retained their typical coiled morphology.

*T. forsythia* did not form mature single-species biofilms in the flow cell system, although it grew well in the growth medium as a planktonic culture. Furthermore, there was no significant difference in biometric parameters of double-species biofilms formed by *P. gingivalis* and *T. denticola* when compared with polymicrobial biofilms formed by the three species (data not shown).

### Gingipains are Essential for Mature Biofilm Formation and the Interaction Between *P. gingivalis* and *T. denticola*

**Figure 3:**
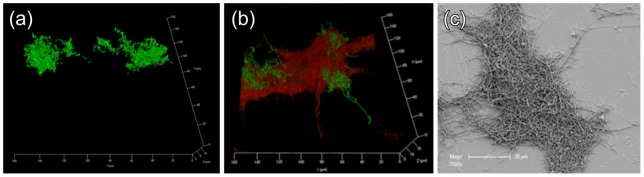
*P.* *gingivalis* W50ABK biofilm development. (a) 3D rendered confocal fluorescence image of *P. gingivalis* W50ABK homotypic biofilm stained with Syto 9 DNA dye. (b) 3D rendered confocal fluorescence image of a representative polymicrobial biofilm containing *P. gingivalis* W50ABK, *T. denticola* and *T. forsythia*, harvested at 90 h. Bacterial cells were stained with species-specific FISH probes (red, *T. denticola*; green, *P. gingivalis*). (c) SEM image of a representative gold-coated *P. gingivalis* W50ABK, *T. denticola* and *T. forsythia* polymicrobial biofilm.

The *P. gingivalis* W50ABK mutant, lacking functional cell surface-located RgpA, RgpB and Kgp gingipains, was used in this study to investigate the role of gingipains in polymicrobial biofilm formation. *P. gingivalis* W50ABK was not able to form mature biofilms after 90 h incubation, with only a few thin clumps of cells up to 12 µm in depth adhering to the glass substratum (Figure 3a). Few cells were detected using SEM, suggesting that the attachment of *P. gingivalis* W50ABK to the glass substratum was not strong enough to withstand the extensive sample preparation required for SEM. When *P. gingivalis* W50ABK was incubated with *T. denticola* and *T. forsythia*, W50ABK was present in low numbers, similar to what was observed in single-species biofilm of the W50ABK mutant. It was not possible to accurately enumerate *P. gingivalis* W50ABK in the polymicrobial biofilm due to the extremely low number of cells. The main component of the polymicrobial biofilm was *T. denticola* and the biofilm structure was similar to *T. denticola* single-species biofilm, except for a small amount of *P. gingivalis* W50ABK associated with *T. denticola* microcolonies (Figure 3b). When compared with single-species *T. denticola* biofilms, there was no significant difference in biometric parameters of *T. denticola* in the polymicrobial biofilms with *P. gingivalis* W50ABK and *T. forsythia*, as determined from five CLSM images at random positions from two biological replicates (Table 1), thus indicating that there was no synergy in biofilm formation between *P. gingivalis* W50ABK and *T. denticola*.

### Biofilm Surface Structure Revealed by Scanning Electron Microscopy

**Figure 4:**
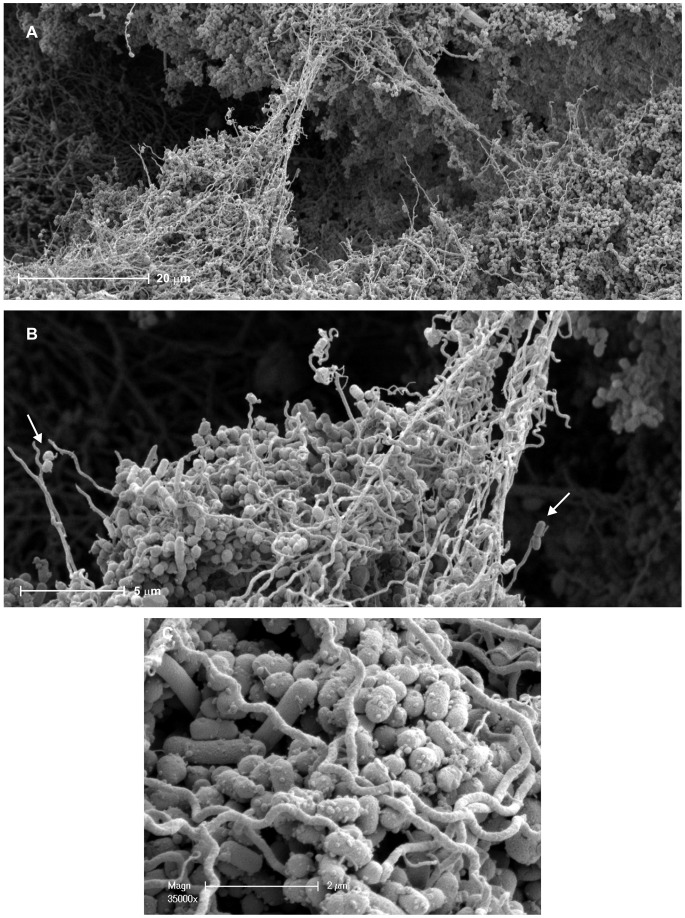
SEM micrographs of polymicrobial biofilms involving wild-type *P. gingivalis*, *T. denticola* and *T. forsythia.* * T. dencicola*, the long thin spirochaete; *P. gingivalis*, the smaller grape-like coccobacillus and *T. forsythia* the larger fusiform rod. Panels B and C are higher magnifications of sections in Panel A. (a) *T. denticola* forming bridges. (b) *P. gingivalis* attached to the ends of *T. denticola* projecting out of the microcolonies. (c) Outer membrane vesicles on the surface of *P. gingivalis.*

**Figure 5:**
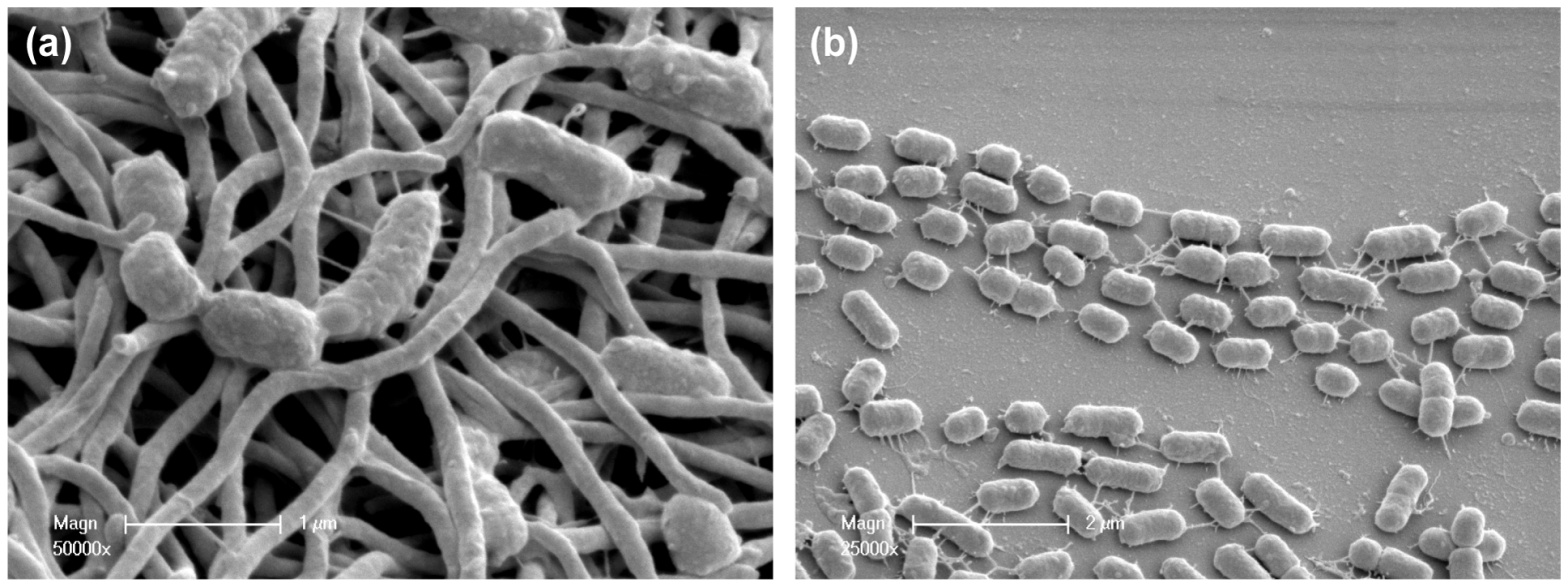
SEM micrographs of polymicrobial biofilms involving *P. gingivalis* W50ABK mutant, *T. denticola* and *T. forsythia*. The left pannel shows *P. gingivalis* W50ABK mutant cells associated with a *T. denticola* microcolony and the right pannel shows *P. gingivalis* cells at the outer edge of a *T. denticola* microcolony. It has been confirmed by FISH that no *T. forsythia* was detected in these biofilms (data not shown).

SEM was used to examine topographies of polymicrobial biofilms with high magnification. On the surface of polymicrobial microcolonies formed by wild-type *P. gingivalis*, *T. denticola* and *T. forsythia*, *P. gingivalis* was the major component (Figure 4). *T. denticola* was closely associated with *P. gingivalis* and showed the typical spiral morphology characteristic of spirochetes (Figure 4). *T. denticola* cells on the surface of microcolonies connected distant *P. gingivalis* cells (Figure 4a). *P. gingivalis* cells were found attached to the ends of *T. denticola* cells that projected out from the surface of the microcolonies (Figure 4b, arrows). A large number of outer membrane vesicles (OMVs) were observed on the surface of wild-type *P. gingivalis* cells in the polymicrobial biofilm (Figure 4c). In *P. gingivalis* homotypic biofilms, OMVs were also observed on the cell surface with a similar abundance. When *P. gingivalis* W50ABK was co-inoculated with *T. denticola* and *T. forsythia*, the majority of cells in biofilms were found to be *T. denticola,* with a few *P. gingivalis* cells either associated within the microcolony (Figure 5a), or at the outer edges of the microcolonies (Figure 5b). The surface of the *P. gingivalis* W50ABK mutant was rough and the shape of the cells less symmetrical (Figure 5a) when compared with wild-type (Figure 4c). Most of the *T. denticola* cells incubated with the *P. gingivalis* W50ABK mutant lost the typical spiral morphology (Figure 5a). Blebbing of the outer membrane of *P. gingivalis* W50ABK mutant cells was not as abundant as that found with wild type (Figure 4c). Furthermore, the blebbing of the mutant exhibited morphology that was distinct from the well-formed OMVs found on wild-type *P. gingivalis* cells (Figures 4c and 5a). Interestingly, filamentous structures were found on *P. gingivalis* W50ABK mutant cells in polymicrobial biofilms formed with *T. denticola* and *T. forsythia*, connecting individual *P. gingivalis* cells (Figure 5b) or between *P. gingivalis* and *T. denticola* (Figure 5a).

## Discussion

Due to the wide-spread use of static biofilm assays on polystyrene surface, *P. gingivalis* W50, which generally adheres poorly to substratum, has been considered as a poor biofilm former [21], [22]. However, it has been suggested that *P. gingivalis* W50-like strains are more virulent in experimental infections and cause a more invasive type of infection when compared with *P. gingivalis* 381/ATCC 33277-like strains, which cause a more localised infection [30]–[32]. By using a dynamic system with a low flow rate designed to mimic the flow of gingival crevicular fluid within the periodontal pocket, we showed that *P. gingivalis* W50 was in fact able to form extensive, mature homotypic biofilms.

*T. denticola* also formed homotypic biofilms, however, the cells in biofilms did not display the typical spiral morphology. Obtaining a model system for the growth of *T. denticola* biofilms has proven to be problematic using static systems [33], but recently was achieved using a continuous culture system. The *T. denticola* cells comprising the homotypic biofilm grown on a coverslip in the continuous culture system displayed the typical spirochete spiral cell morphology [25]. The difference observed in the flow cell system may result from differences in the two model systems. In continuous culture vessels, the substratum for biofilm growth is immersed in liquid culture and in constant contact with planktonic cells, which may alter biofilm growth. The morphology change of *T. denticola* observed in the flow cell model may be related to restricted activities of periplasmic flagella, and as a consequence, limited motility. The morphology of spirochetes is the result of complex interactions between the cell cylinder and the internal periplasmic flagella [34], [35]. In most spirochetes, the periplasmic flagella consist of at least three core proteins (FlaB1, FlaB2 and FlaB3) and a sheath of FlaA protein. It has been shown that FlaB proteins may have effects on the morphology of the periplasmic flagella, since a study reported that mutants of the spirochete *Borrelia burgdorferi* lacking combinations of FlaB and FlaA proteins were completely nonspiral and nonmotile [36]–[38]. We have previously shown that growth of *T. denticola* in a polymicrobial biofilm with *P. gingivalis* resulted in alterations to the abundance of some flagella components. The FlaB protein, TDE1004, increased considerably in coculture, whilst the FlaA proteins, TDE1408 and TDE1409, decreased in abundance, and TDE1712 increased [26].

The extremely low abundance of *T. forsythia* in the polymicrobial biofilm and the finding that *P. gingivalis* and *T. denticola* double-species biofilm showed a similar synergistic effect when compared with three-species biofilm suggests that the *T. forsythia* does not interact closely with *P. gingivalis* and *T. denticola*. This is consistent with a recent study on oral biofilms on natural teeth showing that *T. forsythia* was located in a different layer of subgingival plaque than *P. gingivalis* and *T. denticola* [10].

*P. gingivalis* and *T. denticola* are commonly detected together in the superficial layers of subgingival plaque associated with a chronic periodontitis lesion [9] and synergy has been demonstrated in murine models of periodontitis [28], [39]. Synergistic biofilm formation by *P. gingivalis* strain ATCC 33277 and *T. denticola* has been shown using static assays [17], [18], but *P. gingivalis* W50 failed to form synergistic biofilms with *T. denticola* in the assays [17]. However the findings of this current study demonstrate that *P. gingivalis* W50 interacts with *T. denticola* and strongly participates in polymicrobial biofilm formation. The strong synergy between *P. gingivalis* W50 and *T. denticola* is reflected by the significant increase in biomass, as well as the restored spiral morphology of *T. denticola* in polymicrobial biofilms. SEM images showed that in mature polymicrobial biofilms, *T. denticola* cells form bridges connecting distant cells (Figure 4a). The role of motile bacteria such as *T. denticola* in polymicrobial biofilm development is not well understood. It has been recently shown that motile bacteria can create pores in the biofilm matrix, resulting in an increase in nutrient flow [40]. *T. denticola* is able to move in highly viscous environments [41] and it is possible that in polymicrobial biofilms, *T. denticola* mediates the remodelling of biofilm structure and that the resultant enhanced nutrient flow enables a higher biofilm biomass to be sustained. A *T. denticola* flagella hook *flgE* mutant formed polymicrobial biofilms with *P. gingivalis*, but was only found in the basal layer of the polymicrobial biofilms and no synergy was observed (unpublished data). This suggests that the motility of *T. denticola* plays an important role in synergistic biofilm formation with *P. gingivalis*.

The mature polymicrobial biofilm consisted predominantly of *P. gingivalis*, which has previously been shown to be abundant in subgingival plaque from deep periodontal pockets and the level of which, once above a certain threshold, was predictive of disease progression [42]. In our previous study [26], it was shown that only a few cells of *P. gingivalis* W50 were attached to the glass substratum after the commencement of a constant flow, but the bacterium proliferated quickly after 24 h. *P. gingivalis* coaggregates with *T. denticola* [43], [44] and it has been shown that a *P. gingivalis* gingipain-null mutant lost the ability to coaggregate with *T. denticola* [20]. Our current study shows that gingipains are essential for interaction between *P. gingivalis* and *T. denticola* since the *P. gingivalis* W50ABK mutant had no impact on *T. denticola* biomass and morphology.

Notably, in polymicrobial biofilms formed by wild-type *P. gingivalis*, *T. denticola* and *T. forsythia*, a large number of OMVs were found on the surface of *P. gingivalis* cells. It has been suggested that OMVs plays an important role in intercellular communication and are involved in biofilm formation and virulence of other bacterial species [45]–[47]. A recent study of *Pseudomonas putida* showed that the formation of OMVs leads to an increase in cell surface hydrophobicity, making cells attach more easily to each other as well as the substratum, thus enhancing their ability to form biofilms [48]. It has been suggested that *P. gingivalis* selectively sorts outer membrane proteins into OMVs, resulting in an enrichment of gingipains in OMVs [49]. Besides the catalytic domain, gingipains also encode non-catalytic adhesin domains and these adhesins have been shown to be responsible for the interaction of *P. gingivalis* with other bacteria, including *T. denticola* [20], [50], [51]. It is possible that this enrichment of adhesins in OMVs contributed to the synergistic biofilm formation by *P. gingivalis* and *T. denticola*. In polymicrobial biofilms formed by *P. gingivalis* W50ABK, *T. denticola* and *T. forsythia*, some blebbing of the outer membrane of the W50ABK mutant was observed. However, it appeared that it represented only the initial stages of vesiculation since not many well-formed OMVs were observed on the surface of the mutant compared with wild-type cells. This finding may suggest that gingipains facilitate the maturation of *P. gingivalis* OMVs. The inability of *P. gingivalis* W50ABK to produce OMVs and to form mature homotypic *P. gingivalis* biofilms and form synergistic biofilms with *T. denticola* may suggest that OMVs play a role in *P. gingivalis* biofilm formation or that biofilm formation is an important trigger for OMV production. Interestingly, in polymicrobial biofilms with the W50ABK mutant, an enrichment of filamentous appendages was observed. The filamentous structures connected adjacent *P. gingivalis* cells or *P. gingivalis* with *T. denticola* cells. It is possible that in the absence of gingipains in the *P. gingivalis* W50ABK mutant, other pathways (e.g., minor fimbriae production) are upregulated to compensate for the loss of cell surface adhesins and OMVs.

Taken together, our investigation indicates that there is a strong synergy in biofilm formation between *P. gingivalis* and *T. denticola*. *P. gingivalis* gingipains play important roles in the interaction of the two species as well as in biogenesis of *P. gingivalis* OMVs. It also suggests that multiple pathways may be involved in intercellular interaction in *P. gingivalis* biofilm formation, including OMV production.
